# Supplementary material for: Modeling glioblastoma heterogeneity as a dynamic network of cell states
Source: Mol Syst Biol. 2021 Sep 16;17(9):e10105. doi: 10.15252/msb.202010105 (PMC8444284; doi:10.15252/msb.202010105)
Supplement: Supplementary file 5 — Source Data for Figure 3 [file MSB-17-e10105-s001.zip › Figure3A_sourcedata/GSEA_3065/nosigdb_state1.GseaPreranked.1622718409535/LARSSON_STATE_2.html]

Details for gene set LARSSON\_STATE\_2[GSEA]

|  || Dataset | state1 |
| Phenotype | NoPhenotypeAvailable |
| Upregulated in class | na\_neg |
| GeneSet | LARSSON\_STATE\_2 |
| Enrichment Score (ES) | -0.94518715 |
| Normalized Enrichment Score (NES) | -3.2483137 |
| Nominal p-value | 0.0 |
| FDR q-value | 0.0 |
| FWER p-Value | 0.0 |
Table: GSEA Results Summary

  

Fig 1: Enrichment plot: LARSSON\_STATE\_2      
 Profile of the Running ES Score & Positions of GeneSet Members on the Rank Ordered List

  

| PROBE | GENE SYMBOL | GENE\_TITLE | RANK IN GENE LIST | RANK METRIC SCORE | RUNNING ES | CORE ENRICHMENT || 1 | LMAN1 |  |  | 9094 | -0.151 | -0.9245 | No |
| 2 | CD63 |  |  | 9126 | -0.154 | -0.9236 | No |
| 3 | ACSL3 |  |  | 9338 | -0.187 | -0.9403 | Yes |
| 4 | HSP90B1 |  |  | 9365 | -0.193 | -0.9379 | Yes |
| 5 | MSMO1 |  |  | 9391 | -0.199 | -0.9353 | Yes |
| 6 | CALU |  |  | 9418 | -0.205 | -0.9327 | Yes |
| 7 | SSR4 |  |  | 9449 | -0.213 | -0.9302 | Yes |
| 8 | PRDX4 |  |  | 9457 | -0.215 | -0.9253 | Yes |
| 9 | DDOST |  |  | 9461 | -0.217 | -0.9200 | Yes |
| 10 | HSPA5 |  |  | 9533 | -0.234 | -0.9211 | Yes |
| 11 | CALR |  |  | 9537 | -0.235 | -0.9153 | Yes |
| 12 | PCSK7 |  |  | 9541 | -0.236 | -0.9095 | Yes |
| 13 | ZNF75A |  |  | 9550 | -0.239 | -0.9041 | Yes |
| 14 | DDX39B |  |  | 9552 | -0.241 | -0.8980 | Yes |
| 15 | LAPTM4A |  |  | 9553 | -0.241 | -0.8917 | Yes |
| 16 | TTC14 |  |  | 9578 | -0.252 | -0.8876 | Yes |
| 17 | TMEM259 |  |  | 9603 | -0.260 | -0.8833 | Yes |
| 18 | TNC |  |  | 9605 | -0.262 | -0.8766 | Yes |
| 19 | FTX |  |  | 9617 | -0.265 | -0.8708 | Yes |
| 20 | LENG8 |  |  | 9622 | -0.266 | -0.8643 | Yes |
| 21 | ANKRD11 |  |  | 9637 | -0.275 | -0.8585 | Yes |
| 22 | HMGCR |  |  | 9640 | -0.277 | -0.8515 | Yes |
| 23 | XRCC2 |  |  | 9653 | -0.284 | -0.8454 | Yes |
| 24 | LUC7L3 |  |  | 9667 | -0.292 | -0.8391 | Yes |
| 25 | PDIA3 |  |  | 9687 | -0.301 | -0.8332 | Yes |
| 26 | LRPAP1 |  |  | 9691 | -0.305 | -0.8256 | Yes |
| 27 | HACD3 |  |  | 9697 | -0.311 | -0.8180 | Yes |
| 28 | CREBZF |  |  | 9700 | -0.314 | -0.8101 | Yes |
| 29 | PDIA6 |  |  | 9701 | -0.314 | -0.8019 | Yes |
| 30 | MIR99AHG |  |  | 9706 | -0.321 | -0.7940 | Yes |
| 31 | TMBIM6 |  |  | 9716 | -0.328 | -0.7864 | Yes |
| 32 | ZNF431 |  |  | 9720 | -0.329 | -0.7781 | Yes |
| 33 | ZNF708 |  |  | 9723 | -0.334 | -0.7696 | Yes |
| 34 | MIAT |  |  | 9732 | -0.341 | -0.7616 | Yes |
| 35 | SGCE |  |  | 9733 | -0.341 | -0.7527 | Yes |
| 36 | SF1 |  |  | 9735 | -0.344 | -0.7438 | Yes |
| 37 | APLP2 |  |  | 9736 | -0.344 | -0.7349 | Yes |
| 38 | MARCH6 |  |  | 9740 | -0.350 | -0.7261 | Yes |
| 39 | SPCS2 |  |  | 9744 | -0.353 | -0.7172 | Yes |
| 40 | PRH1 |  |  | 9747 | -0.355 | -0.7082 | Yes |
| 41 | GPC1 |  |  | 9751 | -0.356 | -0.6992 | Yes |
| 42 | SPPL2B |  |  | 9752 | -0.357 | -0.6899 | Yes |
| 43 | RCN2 |  |  | 9759 | -0.364 | -0.6811 | Yes |
| 44 | NKTR |  |  | 9767 | -0.376 | -0.6720 | Yes |
| 45 | ARGLU1 |  |  | 9773 | -0.383 | -0.6626 | Yes |
| 46 | FUS |  |  | 9779 | -0.389 | -0.6529 | Yes |
| 47 | ATP6AP2 |  |  | 9783 | -0.396 | -0.6429 | Yes |
| 48 | COL4A2 |  |  | 9786 | -0.402 | -0.6327 | Yes |
| 49 | MTRNR2L12 |  |  | 9787 | -0.402 | -0.6222 | Yes |
| 50 | DDX17 |  |  | 9788 | -0.405 | -0.6117 | Yes |
| 51 | SQLE |  |  | 9790 | -0.408 | -0.6012 | Yes |
| 52 | B2M |  |  | 9791 | -0.408 | -0.5906 | Yes |
| 53 | LAMP2 |  |  | 9796 | -0.419 | -0.5801 | Yes |
| 54 | KANSL1 |  |  | 9797 | -0.421 | -0.5691 | Yes |
| 55 | CANX |  |  | 9804 | -0.431 | -0.5585 | Yes |
| 56 | VMP1 |  |  | 9817 | -0.489 | -0.5470 | Yes |
| 57 | APP |  |  | 9819 | -0.495 | -0.5342 | Yes |
| 58 | GPM6A |  |  | 9820 | -0.499 | -0.5213 | Yes |
| 59 | SLC1A3 |  |  | 9823 | -0.509 | -0.5082 | Yes |
| 60 | PGRMC1 |  |  | 9826 | -0.516 | -0.4950 | Yes |
| 61 | DDR1 |  |  | 9829 | -0.524 | -0.4816 | Yes |
| 62 | CD9 |  |  | 9832 | -0.544 | -0.4676 | Yes |
| 63 | PLAT |  |  | 9833 | -0.550 | -0.4533 | Yes |
| 64 | COL4A1 |  |  | 9834 | -0.552 | -0.4389 | Yes |
| 65 | PON2 |  |  | 9835 | -0.554 | -0.4245 | Yes |
| 66 | WSB1 |  |  | 9836 | -0.554 | -0.4101 | Yes |
| 67 | LAPTM4B |  |  | 9840 | -0.579 | -0.3954 | Yes |
| 68 | MDM4 |  |  | 9841 | -0.601 | -0.3797 | Yes |
| 69 | ADGRV1 |  |  | 9843 | -0.626 | -0.3635 | Yes |
| 70 | GRIA2 |  |  | 9846 | -0.638 | -0.3471 | Yes |
| 71 | FGFBP3 |  |  | 9850 | -0.651 | -0.3305 | Yes |
| 72 | ADGRG1 |  |  | 9851 | -0.654 | -0.3135 | Yes |
| 73 | NOVA1 |  |  | 9858 | -0.741 | -0.2949 | Yes |
| 74 | MEG3 |  |  | 9859 | -0.749 | -0.2754 | Yes |
| 75 | KCNQ1OT1 |  |  | 9861 | -0.751 | -0.2559 | Yes |
| 76 | NCAM1 |  |  | 9862 | -0.751 | -0.2364 | Yes |
| 77 | SOX6 |  |  | 9863 | -0.798 | -0.2156 | Yes |
| 78 | NEAT1 |  |  | 9866 | -0.816 | -0.1946 | Yes |
| 79 | SNTG1 |  |  | 9867 | -0.828 | -0.1731 | Yes |
| 80 | MALAT1 |  |  | 9868 | -0.912 | -0.1493 | Yes |
| 81 | PTPRZ1 |  |  | 9869 | -0.918 | -0.1255 | Yes |
| 82 | GPM6B |  |  | 9873 | -1.091 | -0.0974 | Yes |
| 83 | PTN |  |  | 9874 | -1.107 | -0.0686 | Yes |
| 84 | BCAN |  |  | 9878 | -1.175 | -0.0383 | Yes |
| 85 | SCRG1 |  |  | 9879 | -1.473 | -0.0000 | Yes |
Table: GSEA details [plain text format]

  

Fig 2: LARSSON\_STATE\_2: Random ES distribution      
 Gene set null distribution of ES for **LARSSON\_STATE\_2**
